# THE RISK OF COVID-19-RELATED MYOCARDITIS IN OLDER ADULTS WITH A HISTORY OF CORONARY ARTERY DISEASE

**DOI:** 10.1093/geroni/igac059.2970

**Published:** 2022-12-20

**Authors:** Andrew Crawford, Ali Vaeli Zadeh, Andres Redondo, Karen Bravo-Ramirez, Ana De, Diego Diaz, Ricardo Criado Carrero, Elias Collado, Joshua Larned

**Affiliations:** Holy Cross Health-University of Miami, Fort Lauderdale, Florida, United States; Holy Cross Health-Jim Moran Cardiovascular Research Institute, Fort Lauderdale, Florida, United States; University of Miami, Fort Lauderdale, Florida, United States; University of Miami at Holy Cross, Miami, Florida, United States; Holy Cross Health-University of Miami, Fort Lauderdale, Florida, United States; University of Miami/Holy Cross Hospital, Fort Luderdale, Florida, United States; University of Miami/Holy Cross Hospital, Fort Lauderdale, Florida, United States; Holy Cross Health-Jim Moran Cardiovascular Research Institute, Fort Lauderdale, Florida, United States; Holy Cross Health- University of Miami, Miami, Florida, United States

## Abstract

**Background:**

Data suggest an increased incidence of myocarditis (MC) associated with the COVID-19 virus. However, the risk factors for COVID-19-related MC remains poorly understood and debated. Therefore, we sought to evaluate the correlation of a history of coronary artery disease (CAD) with MC in older adults admitted for COVID-19.

**Methods:**

Data were obtained from the PearlDiver database (PearlDiver Technologies, Fort Wayne, IN). The study included patients aged 65–75, hospitalized with a primary diagnosis of COVID-19, and Elixhauser Comorbidity index(ECI) >4. History of CAD upon admission was used to split the cohort into two propensity score-matched groups considering age, gender, other cardiovascular diseases, and ECI. Records from both groups were reviewed to identify patients diagnosed with MC during and up to one month after admission. Pearson’s chi-squared test was used to compare groups. The strength of association was reported using Risk Ratios (RR). A p-value < 0.05 was deemed significant.

**Results:**

182,556 patients with and 218,729 without a history of CAD admitted for COVID-19 were identified. Patients with a history of CAD were more likely to be male(54.7% vs. 42% p < 0.0001), older(mean age 70.62 vs. 70.30, p < 0.001), and had more comorbidities(ECI=11 vs. 8, p < 0.0001). After propensity score matching, 0.13% of patients with CAD and 0.12% without CAD developed MC within one month of admission(RR= 1.05, CI95%=0.87–1.26, p=0.61).

**Conclusion:**

One month following admission for COVID-19, the risk of MC was not significantly higher in older persons with a history of CAD.

